# Production and Physicochemical Characterization of Analog Rice Obtained from Sago Flour, Mung Bean Flour, and Corn Flour Using Hot Extrusion Technology

**DOI:** 10.3390/foods10123023

**Published:** 2021-12-06

**Authors:** Siswo Sumardiono, Budiyono Budiyono, Heny Kusumayanti, Nada Silvia, Virginia Feren Luthfiani, Heri Cahyono

**Affiliations:** 1 Department of Chemical Engineering, Faculty of Engineering, Universitas Diponegoro, Semarang 50275, Indonesia; budiyono@live.undip.ac.id (B.B.); silvia.nada15@gmail.com (N.S.); virginiaferen20@gmail.com (V.F.L.); hericahyono@che.undip.ac.id (H.C.); 2 Department of Industrial Chemical Engineering, Vocational School, Universitas Diponegoro, Semarang 50239, Indonesia; henykusumayanti@lecturer.undip.ac.id

**Keywords:** analog rice, sago flour, mung bean flour, hot extrusion, corn flour, physicochemical properties, morphology

## Abstract

Extrusion technology allows the preparation of analog rice, an artificial product made of carbohydrate sources other than rice, with characteristics similar to natural rice. In this study, we aimed at determining the effect of composition and temperature on the nutritional content of analog rice obtained using heat extrusion technology. The physical properties and acceptability of the resulting product were also studied. Skim milk, sago, mung bean, and corn flour as well as the binder carboxymethyl cellulose (CMC) were used. The procedure was conducted in four stages: raw-material preparation, formulation, physicochemical evaluation, and sensory property evaluation. The best analog rice formula was established as 50% sago flour, 30% corn flour, 19.2% mung bean flour, 0.4% skim milk, and 0.4% CMC. The panelists’ most preferred rice analog formula was the one with the highest sago starch and skim milk content. The extrusion temperature did not significantly affect the nutrient content. However, it had a considerable impact on the thermal profile and physical properties, such as appearance and granular morphology.

## 1. Introduction

Most of Indonesia’s population depends on rice as the sole staple food. This fact poses a vulnerability to national food security, so an alternative to rice is needed to reduce its high consumption [1]. Food type diversification is a possible solution to fulfill the need for non-rice staple food sources. Therefore, research on non-rice food, such as analog rice, is needed [2,3]. Analog rice can be developed as a potential product from different grain types with or without added functionalities and nutrients [4]. The analog rice or artificial rice is an imitation of rice made from ingredients from tubers and cereals that looks like rice grains. Analog rice could be made from various raw materials using hot extrusion technology [5,6]. Varying the raw material used would produce analog rice with diverse nutritional contents. Therefore, the raw materials must be selected carefully as they determine the nutritional content, as well as the physical and chemical characteristics of the resulting analog rice product [7]. Analog rice is usually made of 50–98% starch or its derivatives, 2–45% enriching ingredients, and 0.1–10% hydrocolloid [8]. Analog rice products are expected to help the national food stability program by reducing rice consumption levels without changing the Indonesian people’s eating tradition, while satisfying their need for carbohydrates [9].

Sago is the main food for certain Eastern Indonesian people, such as Papua, Maluku, and Sulawesi [10]. It is a rich source of carbohydrates that is used as a rice substitute. The cultivated area of sago plants in Indonesia is estimated to be approximately 1.4 million hectares spread in various regions such as Riau, Mentawai Islands, Bengkulu, Sulawesi, and Irian Jaya [11]. The chemical components of sago starch include 0.19–0.25% protein, 0.10–0.13% lipid, 0.26–0.32% fiber, 0.06–0.43% ash, and 24–31% amylose [12]. The high carbohydrate content, up to 90%, allows sago to become an alternative raw material for artificial rice [4]. However, sago’s protein content is low; therefore, it needs to be fortified to increase its nutritional value [12,13,14,15].

The fortification of cereals or legume flour has been implemented as a suitable strategy to supplement the nutritional quality of cereal-based foods and establish new technologies and marketing for staple foods, such as bread, bread products, and pasta [16]. Beans represent an essential source of proteins, and they are highly consumed in Turkey. Beans are an acidic source of complex carbohydrates, proteins, and dietary fibers. They contain high amounts of vitamins and minerals as sources of potential energy. A widely used fortification ingredient is mung bean (*Vigna radiata* L.), which is a source of vitamins, minerals, and essential amino acids, with a nutritional value comparable to soybeans (*Glycine max* L. Merrill) and red beans (*Phaseolus vulgaris* L.) [17,18].

Corn (*Zea mays* ssp.) is another potential candidate crop for alternative food development, as Indonesia is the largest corn producer in Southeast Asia. In Indonesia, corn production also increased significantly from 6.73 to 17.64 million tons between 1990 and 2011, at an average rate of 5.34% per year [19]. In addition to its carbohydrate and protein content, corn also contains fibers at a level high enough to be used as the raw material for fiber-rich foods [20]. Analog fiber-rich rice products could help reduce cholesterol levels, prevent obesity, or be good alternatives for patients with diabetes who need to consume low-calorie carbohydrates [21,22]. Analog rice production technology can be done by granulation, hot extrusion, and cold extrusion methods [3,19].

Several analog rice studies have used the granulation method [19]. However, the characteristics and appearance of analog rice still did not meet the panelist expectations [19]. Extrusion technology is another valuable method for analog rice production. It comprises mixing, heating, varying conditions, and passing designed to develop and produce extrusion products. It ensures several advantages of analog rice production, including large capacity, the occurrence of drainage processes, mixing, heating, stirring, and shaping, which results in an analog rice product with characteristics similar to that of natural rice [23]. Therefore, this study aimed to determine the effect of composition and temperature on the physical properties, nutritional content, and acceptability of analog rice using heat extrusion technology.

## 2. Materials and Methods

### 2.1. Materials

The materials used in this study consisted of sago flour (*Metroxylon sagu*) (supplied by the Alfurqan Tribinatama, Palopo Sulawesi Selatan Indonesia), mung bean flour (*Vigna radiata*) (supplied by the Gasol Organik Co., Cugenang, Indonesia), cornflour (*Zea mays* L.) (Maizenaku, supplied by the Egafood Co., Jakarta, Indonesia), skim milk powder (Prolac, supplied by the Pendairy Co., Butterworth, Malaysia), CMC (by koepoe-koepoe supplied by the Gunacipta Co., Tangerang, Indonesia), glycerol monostearate (GMS) supplied by the Riken Co., Tokyo, Japan palm oil (supplied by the Salim Ivomas Pratama Co., Jakarta, Indonesia), IR64 (Giant, supplied by PT Hero Supermarket Tbk, Tangerang, Indonesia) rice as standard/comparison rice. All chemicals used were at the analytical grade (Pro Analyst Grade; Merck, Darmstadt, Germany).

### 2.2. Hot Moisture Treatment (HMT)

Sago flour, mung bean flour, skim milk powder, and CMC (ratio of ingredients shown in Table 1) with a dry basis of 400 g were dispersed in 400 mL of stirred distilled water before being mixed with 5 g of GMS, 120 g of corn flour, and 45 mL of palm oil for each sample. All the ingredients were mixed for 20 min. The mixture was then wrapped into a cloth and compressed. It was then steamed for 20 min at approximately 80–90 °C.

### 2.3. Analog Rice Production

The dough from the hot moisture treatment process was molded into the shape of rice using a twin extruder equipped with a process temperature controller, assembled in CV Teguh Jaya Teknik, Ungaran, Indonesia. The extrusion temperature was adjusted to the specified variables (extrusion temperature shown in Table 1). The extruded product was dried under room conditions (temperature 30 °C and RH 80%).

### 2.4. Analyses of the Products

The physical analyses of analog rice products included determination of bulk density [24] and cooking time [25]. Analog rice products were also tested for their carbohydrate, protein, fat, crude fiber, water, and ash contents based on the Association of Official Analytical Chemists (AOAC)methods [26]. The morphology of the analog rice was examined using an analytical scanning electron microscope - Energy Dispersive X-Ray (SEM-EDX) JEOL JSM-6510LA, Tokyo, Japan [27]. Differential scanning calorimetry NEXTA STA (Hitachi STA200RV Tokyo, Japan with real-view sample observation) was used to determine its thermal stability.

### 2.5. Hedonic Sensory Test

Hedonic sensory tests were used to determine the best combined results based on the acceptance/panelists’ preference. The tested parameters included the aroma, color, texture, and taste of analog rice [3]. The tests were conducted by 30 panelists (15 men and 15 women aged 19–21 years, all Indonesian, currently pursuing undergraduate education), using a five-point hedonic scale: dislike very much (1), dislike (2), neutral (3), like (4), and like very much (5).

### 2.6. Statistical Analysis

Experimental data are presented as the mean ± standard deviation (mean ± SD). Each examination was carried out in duplicate. We used a one-way analysis of variance (ANOVA) processed by Duncan’s multiple-range assay to handle data obtained from proximate and calcium assays. The hedonic rating test was performed to distinguish the preference level of the panelists in the case of each product. The hedonic rating test outcome was analyzed using one-way ANOVA and processed with Duncan’s multiple range test. The statistical analysis was performed using Microsoft Excel 2016. The principal component analysis (PCA) was carried out using Origin 2019b (9.65) software.

## 3. Results and Discussion

### 3.1. Analysis of the Raw Materials

Table 2 shows the analysis of the analog rice raw materials. Sago flour contains 78.1% carbohydrates, this level is close to the carbohydrate content of IR64 rice [28,29]. IR64 was used as standard/comparison rice because it is the most widely cultivated hybrid rice variety in Asia, including Indonesia. This rice variety is one of the most consumed by Indonesian people, and the absence of aroma (neutral aroma) is one of the characteristics that make it suitable as the standard in this study. The highest protein content was observed in mung bean flour, reaching 22.44% ± 0.08%, as expected.

### 3.2. Composition of Analog Rice

Table 3 shows the proximate composition of the analog rice produced. The difference in the carbohydrate content was influenced by the composition of the raw materials used. The carbohydrate raw material sources used in AR-4 and AR-7 were 60% and 50% of the total raw material weight, respectively. This produced higher carbohydrate levels in AR-7 than in AR-9. The overall carbohydrate levels in the obtained analog rice products were relatively high, due to their content of sago flour, mung bean flour, and corn flour, which are mostly composed of carbohydrates. However, an increase in the ratio of sago flour had a considerable impact on the carbohydrate content of analog rice. As shown in Table 3, AR-1, AR-4, and AR-7 exhibited higher carbohydrate contents than AR-3, AR-6, and AR-9 (low sago starch ratio group). The carbohydrate level of analog rice was higher than that of IR64, which was only 78.10%. Its high carbohydrate content could make analog rice a better carbohydrate source than natural rice.

We observed that an increased percentage of mung bean (AR-3, AR-6, and AR-9) was associated with higher protein content in the analog rice. However, the protein content of the analog rice was lower than that of the commonly available IR64 rice (7.18%). This is attributable to the low percentage of mung bean flour used in the production of analog rice. Despite exhibiting protein content lower than IR64 rice, analog rice is expected to support daily protein intake.

The ratio of skim milk and mung bean flour in AR-6 analog rice was the highest compared to that in other types of analog rice so that AR-6 had a higher fat content. From the fat content result, it could be understood that, overall, the studied analog rice contained more significant amounts of fat than the IR64 rice (containing 0.36%), which was due to the mung bean flour supplementation. Mung beans are legume commodities with a high fat content. Most of these fats contained unsaturated fatty acids, which have good effects on health [30].

The crude fiber content was lower than that of IR64 rice, containing 3.29% of crude fiber. Fiber sources were mainly obtained from mung bean flour, cornstarch, and CMC. CMC, as a soluble fiber added to food, will increase the fiber content [31]. The addition of CMC produced analog rice with lesser nutritional benefit than the addition of other raw materials.

The composition of the raw materials had a low impact on the moisture content of the analog rice. The moisture content in rice analogs was lower than that of IR64 rice (13.88%). Therefore, moisture content was below the threshold suggested for the safe storage of rice (<14%) wet basis (wb). A moisture content < 14% (wb) prevents the growth of mold, therefore moisture below this value is considered fundamental to ensure the safe storage of cereals [20].

The composition of raw materials showed only a minor effect on the ash content of the analog rice. The ash content ranged from 1.310% ± 0.009% to 1.650% ± 0.007%. Analog rice raw materials (sago flour, mung bean flour, skim milk, and CMC) affect the ash content. The ratio of each raw material in analog rice is the most influential factor, which is related to the relationship between the mass balance of analog rice and its constituent components. However, mung bean flour, in addition to the analog rice that was produced with it (i.e., AR-3, AR-6, and AR-9), had the highest ash content. These three compositions contained a high proportion of mung bean flour.

### 3.3. Effect of Extrusion Temperature on Analog Rice Nutrients

Based on the gelatinization temperature of the raw materials, the extrusion process was performed at 50, 60, 70, 80, and 90 °C. Table 4 shows the results of the proximate analysis of the effect of extrusion temperature on analog rice.

The extrusion temperature affected the carbohydrate content. The contents ranged from 80.80% ± 0.50% to 83.02% ± 0.70%. The carbohydrate content of analog rice did not differ significantly with increasing extrusion temperature. The analog rice extruded at low extrusion temperatures (50 and 60 °C) had a higher carbohydrate content than analog rice extruded at high temperatures (70, 80, and 90 °C). This is potentially caused by the low water content in analog rice that is extruded at high temperatures, which means that the percentage of other ingredients including carbohydrates increases. Moreover, the number of amylose and amylopectin molecules indicates high carbohydrate content in analog rice [32].

The extrusion temperature also affected the protein content. The protein content decreased with increasing extrusion temperature, although with a low significance. We distinguished two groups of significant data: low temperature (50 and 60 °C) and high temperature (70, 80, and 90 °C). This is probably attributable to protein denaturation caused by an exceedingly high extrusion temperature [32].

Furthermore, the extrusion temperature affected the fat content. These results ranged from 1.931% ± 0.004% to 2.514% ± 0.003% fat content. High extrusion temperatures were associated with low fat contents. The decrease in fat content was probably caused by the release of fat in liquid form (oil) from the dough, and the tendency of it to adhere to the extruder screw (similar to the phenomenon observed in the extraction process [33,34]). Statistically significant results were observed at high temperatures. Temperatures between 50 and 70 °C yielded nonsignificant results. By contrast, results were significant at temperatures of 80 and 90 °C.

The extrusion temperature affected the crude fiber content. Crude fiber, which is undigestible, in analog rice comes from the total content of cellulose, hemicellulose, lignin, and pentosane−pentosan, which may be found in raw materials [35,36]. Fiber contents ranged from 3.90% ± 0.04% to 4.39% ± 0.02%. Fiber content decreased from 50 to 70 °C. This could be caused by the rupture and decomposition of the cell walls (hemicellulose) in the analog rice during the extrusion process [37]. However, the fiber content increased at 90 °C. This was thought to occur due to the decreased moisture content in analog rice. During the extrusion process, the moisture content of analog rice decreases due to the evaporation process, this fact causes the percentage of other compounds, such as crude fiber content to increase [38].

The extrusion temperature affected the moisture content as well. Moisture contents ranged from 10.65% ± 0.04% to 13.89% ± 0.05%. The moisture content result showed that the increasing extrusion temperature reduced the moisture content in the analog rice. On extrusion at 50 °C, considered a low temperature, the moisture content remained relatively high. At the temperature of 90 °C, some of the moisture evaporated during the extrusion process, leading to reduced moisture content, due to the high temperatures almost reaching a boiling point at atmospheric pressure [39].

The ash content in the analog rice ranged from 1.741% ± 0.007% to 1.980% ± 0.001%. The association between extrusion temperature and ash content was nonsignificant because the same ratio of raw materials was used for each sample. Ash comprises minerals that are typically stable at high temperatures. Therefore, the ash content did not change considerably during the hot extrusion process.

### 3.4. Physical Analysis of the Analog Rice: Bulk Density and Cooking Time

**Figure 1:**
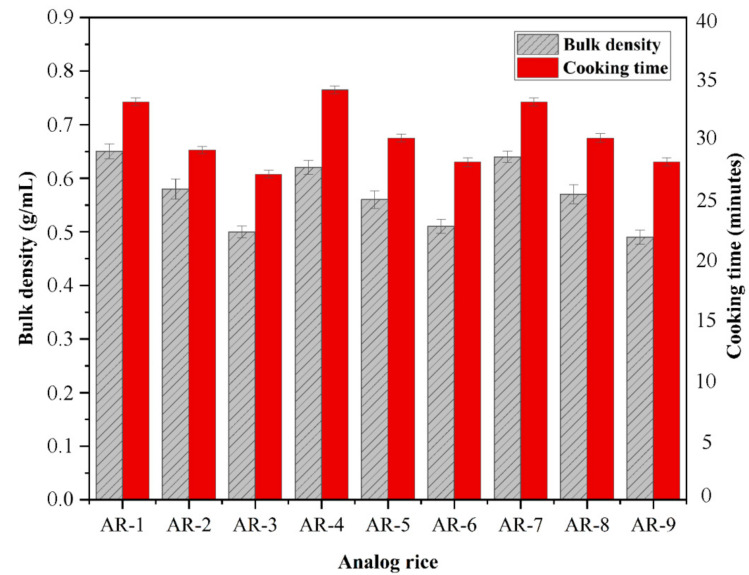
Physical analysis of analog rice composed of different raw materials (at an extrusion temperature of 70 °C). Values are mean ± standard deviation.

Bulk density is defined as the total mass of a material divided by the volume it comprises [40]. Our analog rice exhibited bulk densities ranging from 0.49 ± 0.021 g/mL to 0.65 ± 0.031 g/mL (Figure 1). Analog rice with a high ratio of mung bean flour (AR-3, AR-6, and AR-9) had a lower bulk density than that with a low ratio of mung bean flour (AR-1, AR-4, and AR-7).

**Figure 2:**
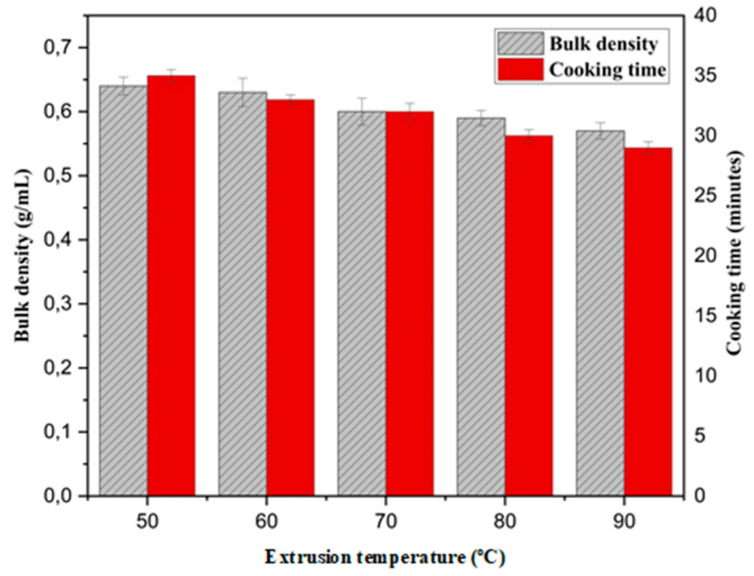
Physical analysis of analog rice based on the temperature difference during the extrusion process. Values are mean ± standard deviation.

An increase in extrusion temperature caused a decrease in the bulk density of analog rice as presented in Figure 2. The highest bulk density at an extrusion temperature of 50 °C was 0.64 ± 0.034 g/mL, and the lowest bulk density at an extrusion temperature of 90 °C was 0.57 ± 0.03 g/mL. This is consistent with the fact that the increase in extrusion temperature caused the water content of the analog rice to decrease. The decrease in water content reduces the mass of analog rice, causing a low bulk density [39]. The average bulk density of our analog rice was 0.599 ± 0.001 g/mL, which is lower than that of IR64 rice (0.79 g/mL) [41].

Based on these results, the analog rice exhibited a lower weight than ordinary rice of the same volume. This result shows that the analog rice’s porosity is higher, influenced by its nutritional content and the manufacturing process, including drying. The drying process makes rice analogs lose water, thus becoming more porous [42].

Cooking time is the time required to cook the rice. The cooking time for analog rice ranged from 29 to 35 min. Raw materials have a considerable effect on cooking time. The longest and shortest cooking times were observed with AR-4 and AR-3, respectively. Analog rice with high sago starch content (AR-1, AR-4, and AR-7) tended to correlate with long cooking time. A higher extrusion temperature was associated with a reduced cooking time. Gelatinization is pivotal for decreasing cooking time [25,43].

The results of the proximate analysis revealed an average crude fiber content of 5.475% ± 0.04%. By contrast, IR64 rice is classified as a product with moderate amylose content and a crude fiber content of 3.29%. During the cooking process, rice expands, which increases the volume of rice but decreases its mass. Therefore, the higher the rice expansion rate is, the lower the bulk density is [44].

### 3.5. Principal Component Analysis

**Figure 3:**
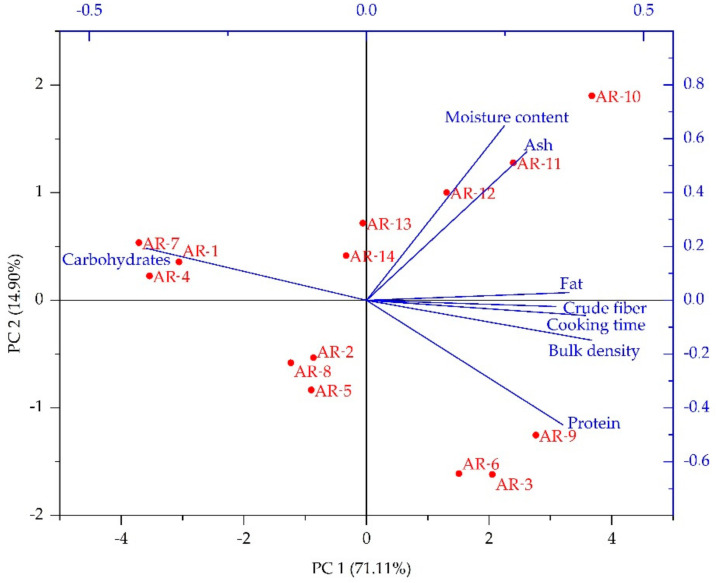
Principal component analysis (PCA) biplot.

The complex multifactor relationship between raw the material ratio and extrusion temperature and the proximate and physical properties of analog rice was explored using principal component analysis (PCA). PCA is a technique used to simplify data, by transforming the data linearly to form a new coordinate system with maximum variance. The PCA biplot is shown in Figure 3 with a total variance of 86.01% with component loads PC1 (71.11%) and PC2 (14.90%).

Figure 3 shows that, in general, all parameters affected the characteristics of the sample. Water content becomes the most influential parameter because it has the vector farthest from the starting point. Meanwhile, crude fiber was the parameter with the lowest effect. Moisture content and ash content had a strong correlation, and fat, crude fiber, cooking time, and density had a relatively larger correlation. AR-1, AR-4, and AR-7 tended to be closer to vector carbides due to their high carbohydrate content derived from sago. Likewise, the protein vectors were approached by AR-6, AR-3, and AR-9. The effect of extrusion temperature greatly affected the water content of analog rice, where AR-10 to AR-14 were in the quadrant where the water content vector was located.

### 3.6. Hedonic Test of Analog Rice

**Figure 4:**
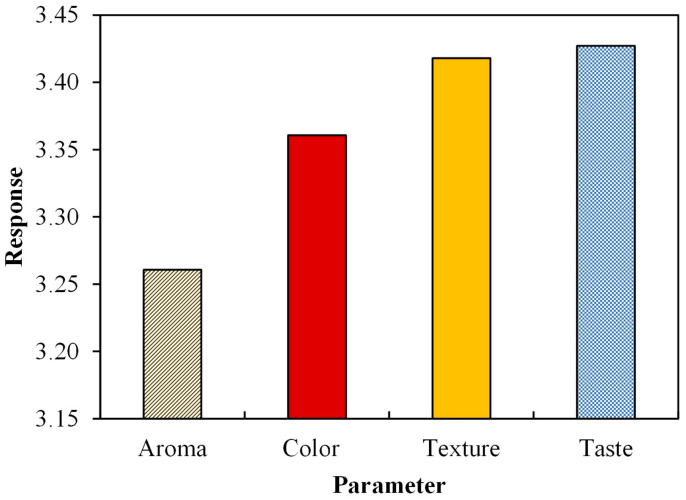
Analysis of respondents’ evaluation of analog rice.

As shown in Figure 4, the respondents rated each parameter of most criteria with a value of 3. This means that they could accept analog rice as a substitute for common rice. The aroma parameter exhibited an average value of 3.26, and the color parameter exhibited an average value of 3.36. According to Sharif et al. [45], in the food industry, aroma or odor testing is considered important as it can quickly provide an assessment of whether a product would be accepted or not. Foodstuffs with an overpowering aroma tend to be less attractive to the public for consumption, especially staple foods such as rice [46,47]. Analog rice aroma is influenced by the extraction process, including the extruder temperature, residence time, moisture content of the raw materials, pressure, or diffusivity of the volatile compounds [48]. The sample aroma was still acceptable for the panelists in analog rice made from sago, mung bean, and corn flour.

Color comes first and is highly decisive in several cases [24,49]. The low amount of fat in skim milk results in a white color. Meanwhile, in the rice sample, the addition of milk provided a slightly lighter color than that of the other samples. This is because skim milk does not contain carotene and riboflavin, which are found in fats [50].

Even if it is considered to be nutritious, delicious, and having a perfect texture, an ingredient would not be eaten if it exhibited an unsightly color or gave the impression that its color deviated from the expectations. Based on the results, the respondents stated that they could accept the analog rice color well. The color produced in this analog rice was brownish yellow, dominated by sago flour, green bean flour, and corn flour.

The texture parameter received an average value of 3.42. These results show that the texture of the analog rice products made with sago flour and corn flour was relatively well accepted. The texture is defined as a property of a food ingredient that the eyes and skin could directly observe and could be determine by chewing in the mouth [51]. Softer analog rice texture is preferable as it imitates the texture of natural rice better. The analog rice texture that could closely resemble the texture of natural rice would exhibit promising potential as a rice substitute [4,47]. The texture is greatly influenced by the level of fineness of the ingredients. The texture of analog rice from sago flour and green beans tends to be smooth and slightly chewy. This is due to the fine texture of the main ingredient in sago flour. Moreover, the milk supplementation makes rice smoother. CMC supplementation also affects the chewiness of analog rice as it hardens the consistency and texture.

The texture and shape of analog rice are also related to the physical processes during its manufacturing. The screw speed during the extrusion process affects analog rice texture and shape. Increasing the screw speed would increase mechanical energy and decrease thermal energy due to the reduced friction between the barrel and the screw and decrease the dough’s residence time in the extruder. Moreover, the non-Teflon (polytetrafluoroethylene)-coated blades on the analog rice cutters tend to form analog rice chains during the cutting process, resulting in imperfect rice grains [52].

The taste parameter received an average rating of 3.37, suggesting that the respondents well accepted the analog rice’s taste. Food is a combination of the various flavors of its ingredients. Taste is the essential aspect that must be met from the various food requirements that exist [53]. Indonesian people who tend to be accustomed to eating rice prefer a rice substitute that has a taste similar to rice in general. Thus, if the analog rice produced has a different taste from rice in general, it is likely to be difficult to accept [54].

**Figure 5:**
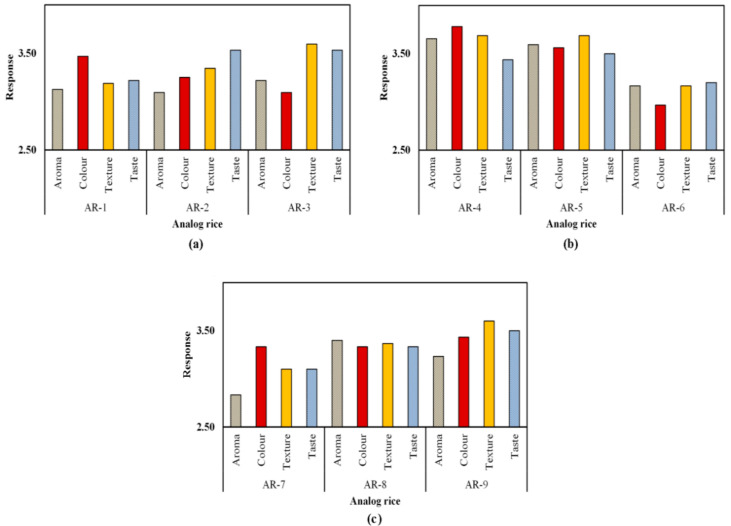
Results of the analog rice hedonic sensory tests (**a**) AR-1; AR-2; AR-3, (**b**) AR-4; AR-5; AR-6, (**c**) AR-7; AR-8; AR-9.

In general, all the analog rice samples in this study received a favorable response from the respondents. Figure 5 presents a detailed analysis of the respondents’ most-preferred and least-preferred analog rice. The best-performing analog rice was AR-4, which contained the highest percentage of sago flour, a low proportion of mung bean flour, and skim milk, but no CMC. By contrast, the least-preferred analog rice was AR-7, which was identical to AR-4 in composition except that AR-7 also contained CMC.

### 3.7. Morphology of Analog Rice

**Figure 6:**
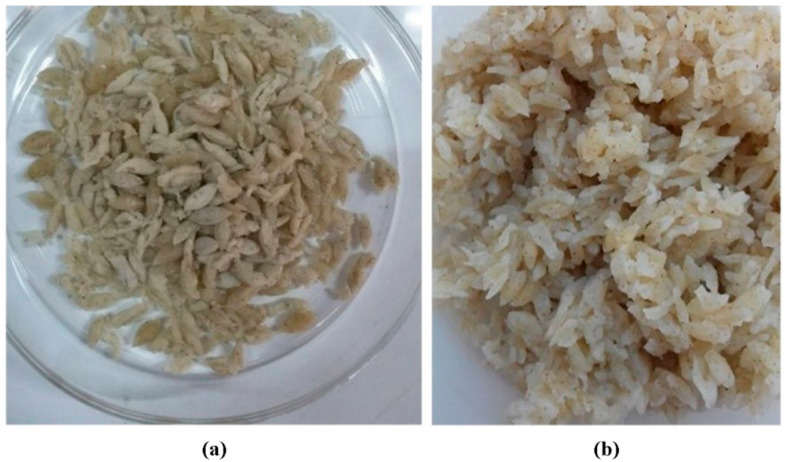
Best analog rice AR-9 (50% sago flour, 19.2% mung bean flour, 30% corn flour, 0.4% carboxymethyl cellulose, and 0.4% skim milk) before (**a**) and after cooking (**b**).

**Figure 7:**
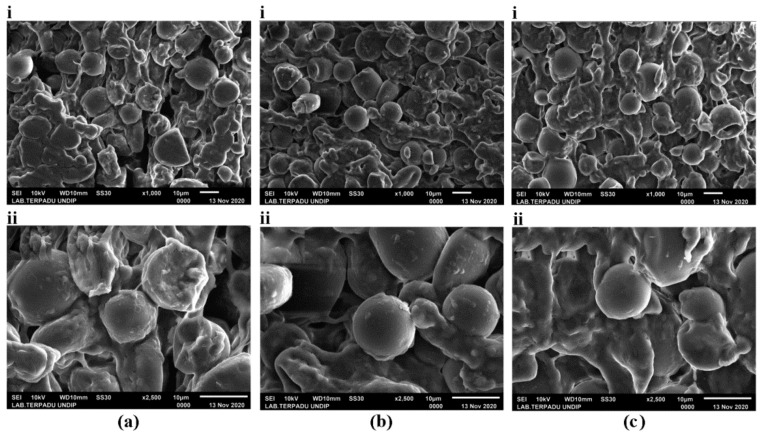
Morphological characteristics of analog rice (**a**) AR-10, (**b**) AR-12, and (**c**) AR-14 under (**i**) 1000× and (**ii**) 2500× magnification.

Based on the proximate compositions, we concluded that AR-9 (Figure 6) was the best analog rice, containing 50%, 19.2%, 30%, 0.4%, and 0.4% sago flour, mung bean flour, corn flour, CMC, and skim milk, respectively. The variations in the morphological appearance of the analog rice according to the extrusion temperature, i.e., 50 °C (AR-10), 70 °C (AR-12), and 90 °C (AR-14), are presented in Figure 7, which depicts intact and broken starch granules. Figure 7a(i,ii) with magnifications of 1000× and 2500×, respectively, reveals gaps between the starch granules in AR-10. By contrast, AR-12 (Figure 7b(i,ii)) and AR-14 (Figure 7c(i,ii)) reveal more adhesion between the starch granules.

The swelling of granules, when mixed with water, and gelatinization during hot extrusion causes the starch granules to break the intramolecular bonds and adhere to each other [55,56,57,58]. The morphological appearance of AR-12 and AR-14 seems similar. However, when observed in detail, AR-14 exhibits a higher granule density than AR-12. The higher the extrusion temperature was, the higher was the number of broken bonds in the starch. Budi et al. (2015) [59] revealed that at a temperature of 70–90 °C, the degree of gelatinization of starch dough for analog rice reached 100%.

### 3.8. Differential Scanning Calorimetry (DSC) Analysis of Analog Rice

**Figure 8:**
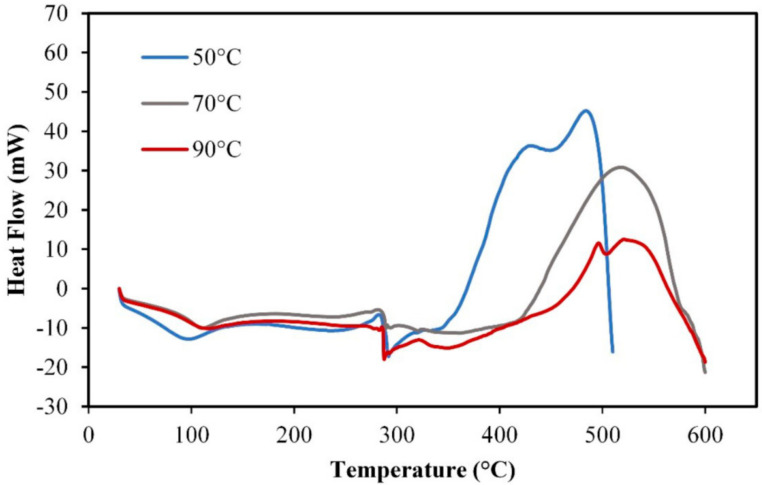
DSC thermogram of analog rice (AR-9) at extrusion temperatures of 80, 85, and 90 °C.

The DSC profile of analog rice produced at temperatures of 50 °C (AR-10), 70 °C (AR-12), and 90 °C (AR-14) is depicted in Figure 8. The use of materials such as sago flour, mung bean flour, skim milk, and CMC caused several peaks that can be seen in the thermogram. At a temperature of approximately 100 °C, an endothermic peak occurred, indicating that the analog rice underwent a change in physical structure. However, at this peak, AR-10 exhibited a lower temperature than AR-12 and AR-14. The exothermic peak occurred at approximately 300 °C, indicating that the analog rice underwent changes in its physical and chemical properties. Furthermore, differences in thermal profiles began to occur at temperatures above 400 °C. The difference in the thermal profile is represented by AR-10, which reached the exothermic peak at a temperature of approximately 470 °C; by contrast, the exothermic peak of AR-12 and AR-14 occurred at approximately 530 °C. This phenomenon is related to the gelatinization of AR-10 that occurred at a lower level than that of other samples. The degree of gelatinization affects the thermal stability of a starchy material [60,61].

## 4. Conclusions

Based on the proximate compositions, we concluded that AR-9 was the best analog rice, containing 50%, 19.2%, 30%, 0.4%, and 0.4% of sago flour, mung bean flour, corn flour, CMC, and skim milk, respectively. This analog rice sample showed the highest protein content, and the carbohydrate levels were sufficiently high to be consumed as a staple food. The extrusion temperature had a significant effect on the physical properties of analog rice, showing that 70 °C was the optimum temperature. The analog rice exhibited a bulk density of 0.599 g/mL and required a cooking time of 29–35 min. Extrusion temperature affected the morphology and thermal profile of analog rice. The texture, aroma, taste, and appearance of analog rice resembled those of rice paddy (IR64 for rice standard); therefore, analog rice can be a substitute for ordinary rice. However, there are still many aspects that could be the object of further research. The addition of nutrients or active substances for specific target panelists and studies on the efficiency in the extrusion process are challenging targets for future investigation.

## Figures and Tables

**Table 1:** Analog rice sample code: raw material ratio difference and extrusion temperature.

Analog Rice Sample Code	Sago Flour (%*w/w*)	Corn Starch (%*w/w*)	Mung Bean Flour (%*w/w*)	Skim Milk (%*w/w*)	CMC (%*w/w*)	Extrusion Temperature (°C)
AR-1	60	30	9.6	-	0.4	70
AR-2	55	30	14.6	-	0.4	70
AR-3	50	30	19.6	-	0.4	70
AR-4	60	30	9.6	0.4	-	70
AR-5	55	30	14.6	0.4	-	70
AR-6	50	30	19.6	0.4	-	70
AR-7	60	30	9.2	0.4	0.4	70
AR-8	55	30	14.2	0.4	0.4	70
AR-9	50	30	19.2	0.4	0.4	70
AR-10	50	30	19.2	0.4	0.4	50
AR-11	50	30	19.2	0.4	0.4	60
AR-12	50	30	19.2	0.4	0.4	70
AR-13	50	30	19.2	0.4	0.4	80
AR-14	50	30	19.2	0.4	0.4	90

CMC, carboxymethyl cellulose.

**Table 2:** Proximate analysis of analog rice raw materials.

Analog Rice Raw Material	Carbohydrates (%)	Protein (%)	Fat (%)	Crude Fiber (%)	Moisture Content (%)	Ash (%)
Sago flour	83.92 ± 0.62 a	0.92 ± 0.02 e	0.994 ± 0.002 d	0.87 ± 0.03 f	6.80 ± 0.05 c	7.370 ± 0.003 c
Bean flour	58.33 ± 0.35 f	22.44 ± 0.08 a	2.693 ± 0.006 a	3.68 ± 0.02 b	7.55 ± 0.05 b	8.994 ± 0.005 b
Cornstarch	80.56 ± 0.73 d	9.11 ± 0.04 b	1.540 ± 0.003 b	5.42 ± 0.04 a	2.99 ± 0.05 e	0.581 ± 0.001 e
Skim milk	84.32 ± 0.58 b	1.36 ± 0.01 d	1.281 ± 0.001 c	1.29 ± 0.02 e	5.09 ± 0.05 d	7.952 ± 0.004 d
CMC	82.96 ± 0.24 c	0.90 ± 0.01 e	1.294 ± 0.002 c	1.65 ± 0.02 d	5.36 ± 0.05 d	9.490 ± 0.006 a
IR64 (rice standard)	78.10 ± 0.12 e	7.18 ± 0.04 c	0.360 ± 0.004 e	3.29 ± 0.04 c	13.88 ± 0.04 a	0.560 ± 0.004 e

Values are mean ± standard deviation. The values followed by distinct letters (a–f) in the same column are statistically significant; *p* < 0.05, *n* = 3.

**Table 3:** Proximate analysis of analog rice with different compositions of raw materials (at an extrusion temperature of 70 °C).

Analog Rice Sample Code	Carbohydrates (%)	Protein (%)	Fat (%)	Crude Fiber (%)	Moisture Content (%)	Ash (%)
AR-1	83.44 ± 0.20 b	2.97 ± 0.03 c	1.671 ± 0.003 d	2.39 ± 0.04 fg	10.55 ± 0.04 a	1.374 ± 0.003 bc
AR-2	82.75 ± 0.30 c	3.88 ± 0.07 b	1.653 ± 0.002 d	3.89 ± 0.05 c	10.21 ± 0.07 b	1.511 ± 0.002 b
AR-3	80.93 ± 0.90 e	4.76 ± 0.07 a	2.552 ± 0.006 a	3.48 ± 0.07 d	10.35 ± 0.03 ab	1.481 ± 0.008 b
AR-4	84.15 ± 0.50 a	2.73 ± 0.06 d	1.561 ± 0.007 de	2.11 ± 0.02 g	10.17 ± 0.07 b	1.310 ± 0.009 c
AR-5	82.50 ± 0.80 cd	3.83 ± 0.02 b	2.081 ± 0.003 c	3.01 ± 0.03 e	10.28 ± 0.06 ab	1.330 ± 0.006 c
AR-6	81.66 ± 0.10 d	4.8 ± 0.01 a	1.981 ± 0.005 cd	4.45 ± 0.01 b	10.28 ± 0.08 ab	1.460 ± 0.009 b
AR-7	84.31 ± 0.40 a	2.66 ± 0.02 d	1.354 ± 0.007 e	2.62 ± 0.05 f	10.33 ± 0.03 ab	1.362 ± 0.005 bc
AR-8	82.63 ± 0.70 c	4.03 ± 0.04 ab	1.702 ± 0.006 d	2.06 ± 0.06 g	10.49 ± 0.04 a	1.452 ± 0.007 b
AR-9	80.73 ± 0.50 e	4.83 ± 0.02 a	2.280 ± 0.003 b	5.57 ± 0.07 a	10.51 ± 0.02 a	1.650 ± 0.007 a

Values are mean ± standard deviation. The values followed by distinct letters (a–g) in the same column are statistically significant; *p* < 0.05, *n* = 3.

**Table 4:** Proximate composition of analog rice as affected by the extrusion temperature.

Analog Rice Sample Code	Extrusion Temperature (°C)	Carbohydrates (%)	Protein (%)	Fat (%)	Crude Fiber (%)	Moisture Content (%)	Ash (%)
AR-10	50	80.80 ± 0.50 b	4.15 ± 0.01 a	2.514 ± 0.003 a	4.38 ± 0.06 a	13.89 ± 0.05 a	1.980 ± 0.001 a
AR-11	60	81.48 ± 0.30 b	4.06 ± 0.04 a	2.262 ± 0.002 a	4.39 ± 0.02 a	13.09 ± 0.02 b	1.810 ± 0.003 b
AR-12	70	82.57 ± 0.20 a	3.71 ± 0.06 b	2.572 ± 0.007 a	3.90 ± 0.04 b	11.07 ± 0.07 c	1.951 ± 0.002 a
AR-13	80	82.67 ± 0.60 a	3.66 ± 0.02 b	1.722 ± 0.002 b	3.96 ± 0.05 b	10.78 ± 0.08 d	1.873 ± 0.005 a
AR-14	90	83.02 ± 0.70 a	3.76 ± 0.04 b	1.931 ± 0.004 b	4.28 ± 0.02 a	10.65 ± 0.04 d	1.741 ± 0.007 b

Values are mean ± standard deviation. The values followed by distinct letters (a–d) in the same column are statistically significant; *p* < 0.05, *n* = 3.

## Data Availability

The datasets generated and/or analyzed during the current study are available from the corresponding author on reasonable request.
